# Towards standardizing mitral transcatheter edge-to-edge repair with deep-learning algorithm: a comprehensive multi-model strategy

**DOI:** 10.3389/fnetp.2025.1701758

**Published:** 2025-11-25

**Authors:** Silvia Corona, Théo Godefroy, Olivier Tastet, Denis Corbin, Thomas Modine, Stephan von Bardeleben, Frédéric Lesage, Walid Ben Ali

**Affiliations:** 1 Structural Heart Valve Center, Montreal Heart Institute, Montreal, QC, Canada; 2 Biomedical Engineering Department, Polytechnique Montréal, Montreal, QC, Canada; 3 UMCV, Hôpital Haut-Lévêque, CHU Bordeaux, Bordeaux, France

**Keywords:** mitral regurgitation, AI, M-TEER, deep-learning, segmentation, TEE, 4D

## Abstract

**Background:**

Severe mitral valve regurgitation requires comprehensive evaluation for optimal treatment. Initial screening uses transthoracic echocardiography (TTE), followed by transesophageal echocardiography (TEE) to determine eligibility for adequate intervention. Mitral Transcatheter Edge-to-Edge Repair (M-TEER) indications are based on detailed and quality valve and sub-valvular apparatus assessment, including anatomy and regurgitation pathophysiology.

**Aim:**

To develop AI algorithms for standardizing M-TEER eligibility assessment using TTE and TEE echocardiograms, supporting all stages of mitral valve regurgitation evaluation to assist non-expert centers throughout the entire process, from severe mitral valve regurgitation diagnostic to M-TEER procedure.

**Methods:**

Three deep learning algorithms were developed using echocardiographic data from M-TEER patients performed at Montreal Heart Institute (2018–2025). 1. ECHO-PREP was trained to identify key diagnostic views in TTE (n = 530) and diagnostic and procedural views in TEE (n = 2,222) examinations to determine the level of quality images needed to do a M-TEER. 2. 4D TEE segmentation with automated mitral valve area (MVA) quantification (n = 221), and 3. 2D TEE scallop-level segmentation of leaflets and sub-valvular structures (n = 992).

**Results:**

Preliminary results on test sets showed 95.7% accuracy in TTE view classification and 91% accuracy for TEE view classification. The 4D segmentation module demonstrated excellent agreement with manual MVA measurements (R = 0.84, p < 0.001), successfully discriminating patients undergoing M-TEER from those referred for surgical replacement (p = 0.046 for AI predictions). The 2D scallop-level analysis achieved a mean Dice score of 0.534 across 11 anatomical structures, with better performance in commonly represented configurations (e.g., A2-P2, P1-A2-P3).

**Conclusion:**

ECHO-PREP demonstrates the feasibility of an integrated AI-assisted workflow for MR assessment, combining quality control, dynamic 4D valve quantification, and scallop-level anatomy interpretation. These results support the potential of AI to standardize M-TEER eligibility, reduce inter-observer variability, and provide decision support across centers with different levels of expertise.

## 1 Introduction

Mitral regurgitation (MR) is a prevalent valvular heart disease, affecting approximately 2% of the general population and up to 10% of individuals over 75 years of age (Nkomo et al., 2006). In patients with severe MR and high or prohibitive surgical risk, transcatheter edge-to-edge repair (M-TEER) has emerged as an established therapeutic option that can reduce symptoms, hospitalizations, and improve quality of life (Stone et al., 2018; Maisano et al., 2013).

Successful M-TEER depends critically on detailed anatomic and functional characterization of the mitral valve apparatus. This complex apparatus is a dynamic interface between the left atrium and ventricle, composed of two leaflets attached to a saddle-shaped annulus and supported by a subvalvular network of chordae tendineae and papillary muscles. Transesophageal echocardiography (TEE) remains the cornerstone imaging modality for pre-procedural assessment and intra-procedural guidance (Zamorano et al., 2011), providing high-quality imaging of cardiac structures in 2D and 3D, enabling real-time dynamic assessment. In contrast, transthoracic echocardiography (TTE) is typically reserved for initial screening and post-procedural follow-up. Precise quantification of valvular morphology and kinematics from these images can also feed into computational models, such as finite element simulations, to replicate patient-specific biomechanics (Votta et al., 2008). Deriving this level of detail, particularly a pixel-wise annotation of valve substructures from 4D TEE data, is a formidable task. The automation of mitral valve segmentation and tracking is hindered by intrinsic challenges of echocardiography, such as artifacts from patient motion, variable image quality, and scarse availability of expertly annotated 4D datasets for training. However, conventional clinical workflows rely heavily on expert interpretation and manual measurements, which are time-consuming and subject to inter- and intra-observer variability (Hien et al., 2014; Thomas et al., 2008). Artificial intelligence (AI), particularly deep learning, offers an opportunity to overcome these limitations by providing rapid, reproducible, and quantitative analysis of echocardiographic images.

Convolutional neural networks (CNNs), particularly encoder-decoder architectures like U-Net and its 3D extensions, have demonstrated remarkable success over the last decade in automating tasks in cardiac ultrasound, including chamber segmentation, functional analysis, and valvular assessment (Leclerc et al., 2019; Ouyang et al., 2020). Clinical and technical precedents illustrate this trajectory. Vendor-integrated solutions such as Anatomic Intelligence in Ultrasound (AIUS) (Philips Healthcare) have implemented automated recognition and measurement of cardiac structures, showing the feasibility of integrating anatomy-aware algorithms into daily workflows. Academic initiatives and challenges (e.g., the Mitral Valve Segmentation challenge -MVSEG- at the International Conference on Medical Image Computing and Computer Assisted Intervention congress -MICCAI-) have provided standardized benchmarks to accelerate innovation and compare algorithmic performance. The winning model at MVSEG 2023 (Synapse, 2025), often leveraging advanced architectures like nnU-Net or vision transformers, achieved state-of-the-art Dice scores, showcasing an unprecedented ability to accurately delineate the thin, dynamic mitral leaflets and complex annular geometry.

Several research groups have contributed to this field. Costa et al. (2019) developed a 2D CNN for leaflet segmentation in 2D TTE, while Carnahan et al. (2021) and Aly et al. (2022) focused on 3D segmentation from TEE using a 3D Residual UNet and nnUNet, respectively. Chen et al. (2023) introduced a two-stage nnUNet approach, initializing it with a classifier pre-trained to identify the valve’s open and closed states. Munafò et al. (2024) created a Multi-Decoder Residual UNet to segment the annulus and both leaflets separately at end-systole from 3D TEE. A critical limitation of these studies is their inability to perform frame-by-frame (4D) analysis of the entire valve apparatus throughout the cardiac cycle. Previous 4D efforts have been restricted to annulus-specific segmentation (Andreassen et al., 2019; Andreassen et al., 2022) or tracking (Taskén et al., 2023), or were confined to 2D imaging for leaflets and annulus, as seen in the work of Wifstad et al. (2024), who used a UNet with attention gates for 2D TTE. Recently, Munafò et al. (2025) proposed a semi-supervised training strategy using pseudo-labeling for MV segmentation in 4D TEE employing a Teacher-Student framework to ensure reliable pseudo-label generation. The Student model demonstrated reliable frame-by-frame MV segmentation on 120 4D TEE recordings from 60 candidates for MV repair, accurately capturing leaflet morphology and dynamics throughout the cardiac cycle, with a significant reduction in inference time compared to the ensemble. Despite these advances, several challenges persist. Generalizability across vendors and imaging protocols is limited, and a fully automated 4D MV segmentation with a scallop-level analysis, which is also able to provide automated measurements in complex anatomies to define the M-TEER eligibility, is difficult. The development of such a method is highly challenged by the labor-intensive manual annotation process needed to generate the extensive datasets required for the supervised training of CNNs.

In this context, we developed an integrated deep learning framework for the comprehensive pre-procedural assessment of the mitral valve in patients with severe mitral regurgitation. Our solution features a three-stage algorithmic pipeline designed to: 1. assess the quality of available TTE and TEE images, 2. perform segmentation of the mitral annulus, leaflets, and scallops, and 3. automatically compute the mitral valve area (MVA) from 4D-TEE volumes. By generating reproducible, clinically relevant measurements, this approach has the potential to standardize feasibility assessment and support heart team decision-making in transcatheter mitral interventions.

## 2 Methods

**FIGURE 1:**
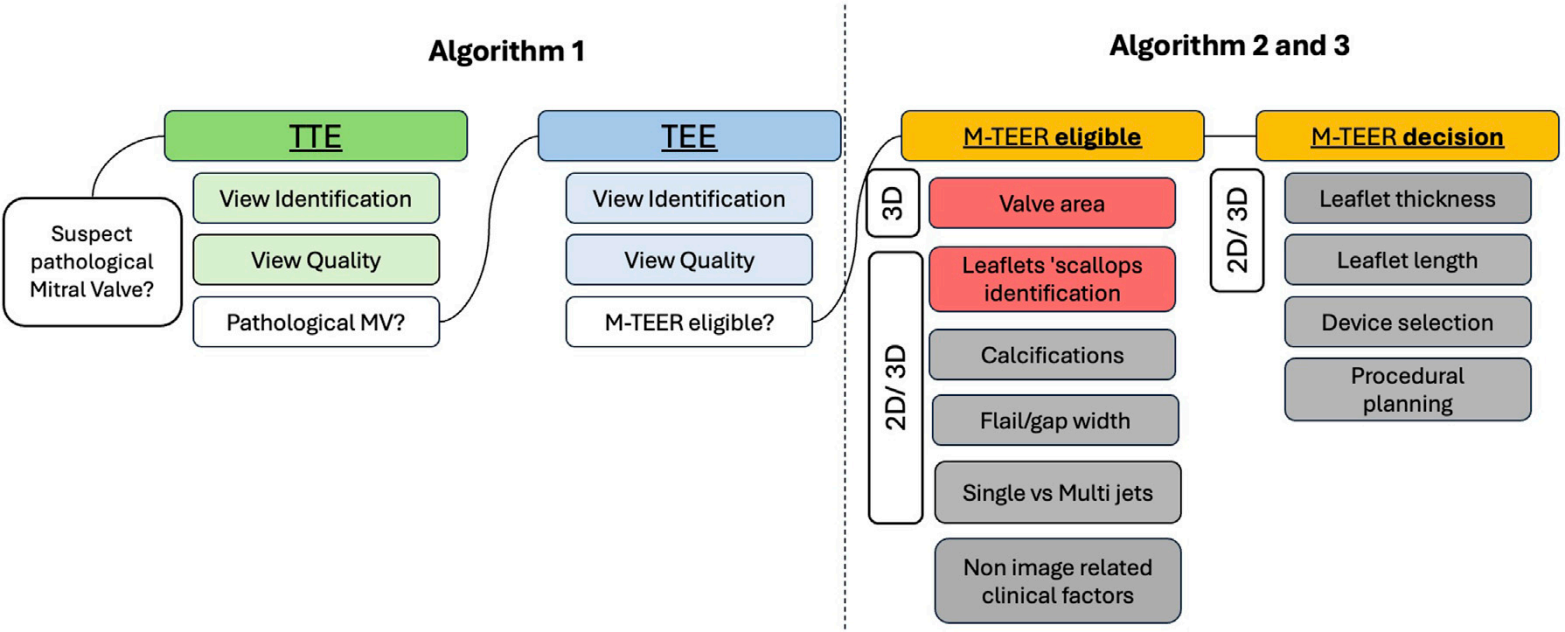
ECHO-PREP fully automatic clinical workflow for MV Assessment and M-TEER procedural planning. Grey boxes denote potential applications of the available algorithms that are currently under development or have not yet been validated. 2D = two-dimensional; 3D = three-dimensional; M-TEER = mitral transcatheter edge-to-edge repair; MV = mitral valve; TTE = transthoracic echocardiography; TEE = transesophageal echocardiography.

The proposed original multi-step workflow, called ECHO-PREP, consisting of three sequential algorithms for image quality assessment, mitral valve segmentation, including scallop-level analysis, and automated measurements, is illustrated in Figure 1.

TTE and TEE pre-procedural images from M-TEER (Mitraclip) and surgical mitral valve replacement (MVR) patients, performed at the Montreal Heart Institute from 1 January 2018, to 1 January 2025, were retrospectively collected. Both two-dimensional (2D) images, three-dimensional (3D), and four-dimensional (4D) volume images were used. 3D refers to single-volume acquisitions, whereas 4D refers to multi-volume datasets spanning the entire cardiac cycle. Our algorithm was primarily trained on 3D echo volumes to establish accurate segmentation performance. Once optimized in this setting, the model was subsequently extended and retrained to analyze sequences of 3D volumes across the cardiac cycle, thereby enabling full 4D assessment.

### 2.1 Automatic classification of 2D- TTE and TEE images: quality views assessment

#### 2.1.1 Dataset processing and splitting

TEE and TTE video images were processed through a systematic pipeline. All frames were extracted from source videos using OpenCV, with each frame inheriting its parent video’s label. Multi-label annotations were transformed to single labels using priority rules, removing technical artifacts such as ‘delivery_system’ and ‘clip’ tags. Dataset splitting was performed at the video level using instance_uid identifiers to prevent data leakage, ensuring no video appeared in multiple splits. Videos were stratified by label distribution and randomly assigned to training (50%), validation (25%), and test (25%) sets. This video-level splitting approach maintained temporal integrity while enabling robust model evaluation.

#### 2.1.2 Model architecture and training

**FIGURE 2:**
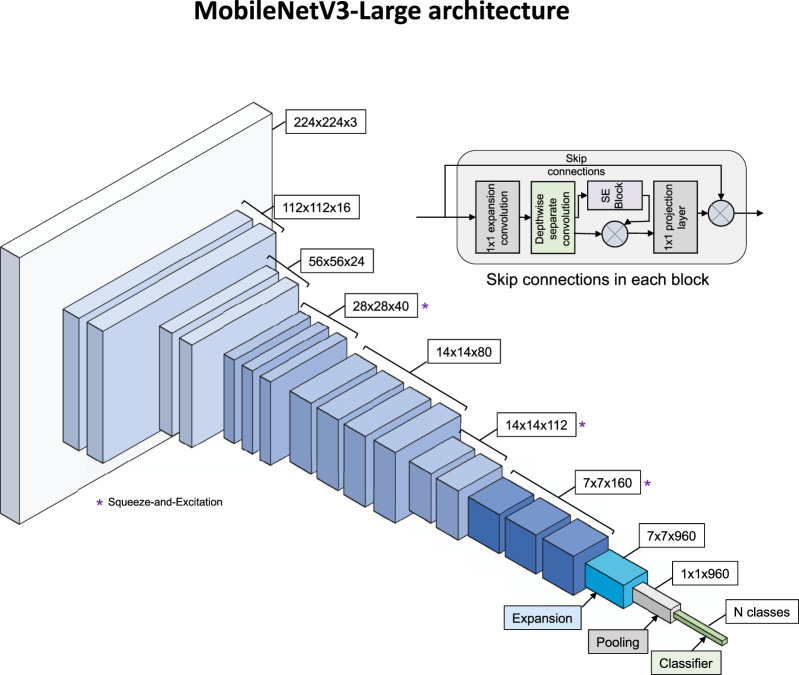
MobileNetV3-Large architecture. Main diagram shows feature map progression from 224×224×3 input through inverted residual blocks to N-class output. Blocks marked (*) use Squeeze-and-Excitation modules. Inset shows internal structure of an inverted residual block with skip connections.

We employed MobileNetV3-Large (Elaziz et al., 2023) as our base architecture, initialized with pre-trained weights from ImageNet (Figure 2). For both quality assessment (binary classification) and view classification (multi-class single-label), only the final classification layer was modified to match the target classes, implementing a transfer learning approach. Training images underwent augmentation, including random horizontal and vertical flips, random rotation (+/−10°), resizing to 256 × 256 pixels, and random cropping to 224 × 224 pixels. Validation and test images underwent deterministic preprocessing, which included resizing to 256 × 256 pixels and center cropping to 224 × 224 pixels. To accelerate training, entire datasets were loaded into memory using a custom Dataset class. Models were trained using the Adam optimizer with a learning rate of 1 × 10^ (−4) and cross-entropy loss for 100 epochs, with a batch size of 128. Training utilized eight parallel data loading workers and CUDA acceleration. Model selection was based on validation accuracy, with the best-performing checkpoint saved for inference. Performance was monitored using both accuracy and the area under the receiver operating characteristic curve (AUROC). AUROC was computed using one-vs-rest methodology for multi-class view classification.

### 2.2 4D-TEE-based automatic MV segmentation and MVA measurements

#### 2.2.1 Dataset and data preparation

This study utilized the MVSEG2023 public dataset (Synapse, 2025), a standardized collection of TEE volumes acquired using the Philips EPIQ cardiac ultrasound system. The dataset contains segmentations for the anterior and posterior leaflets (Labels 1 and 2). To enhance the dataset for comprehensive valve analysis, manual annulus contour segmentations were added (Label 3).

##### 2.2.1.1 Annulus annotation enhancement

Manual annulus contours were created using 3D Slicer by placing control points along the mitral annulus in 3D space using the SlicerHeart analysis module. These control points were exported as JSON markup files containing world coordinates. To convert these sparse control points into volumetric segmentations, an automated spline-based approach was developed: 1. control points were fitted with a smooth 3D B-spline using scipy’s splprep function with zero smoothing factor, 2. the spline was evaluated at 100 equally spaced parameter values to create a dense point cloud, 3. a cylindrical tube with 1.5 mm radius was generated around the spline using VTK libraries, and 4. the tube was voxelized into the original image space using VTK’s vtkPolyDataToImageStencil method.

To ensure anatomically consistent segmentations, we applied morphological post-processing, including connected component analysis, to retain only the most significant component with the highest mean z-coordinate, effectively removing spurious disconnected regions.

#### 2.2.2 Deep learning model training

##### 2.2.2.1 Architecture and framework

We employed MONAI’s Auto3DSeg framework, which automatically generates and optimizes multiple 3D segmentation architectures for medical imaging applications. The framework was configured to use SegResNet as the primary architecture, a 3D residual U-Net variant designed explicitly for volumetric medical image segmentation.

##### 2.2.2.2 Training configuration

The enhanced MVSEG2023 dataset was divided into 5-fold cross-validation splits with random stratification (seed = 42). Training data organization followed MONAI’s standard format. The Auto3DSeg pipeline automatically handled data preprocessing, augmentation strategies, and hyperparameter optimization. The training was set up following the configuration of the MVSEG2023 challenge winner, with the specified modality being magnetic resonance imaging.

##### 2.2.2.3 Model ensemble

The Auto3DSeg pipeline trains a model for each fold and enables ensemble prediction by averaging the outputs of all models, which improves performance at the expense of longer inference time. For prediction, models from all five folds were used to obtain the best segmentation.

#### 2.2.3 Cardiac phase detection and temporal analysis

##### 2.2.3.1 End-systole identification

To identify the optimal cardiac phase for valve area measurement, an automated mid-diastole detection algorithm based on temporal analysis of segmented structures was developed. For each frame in the 4D TEE sequences, we performed the following analysis pipeline:

- Annulus Skeletonization: The segmented annulus (Label 3) was skeletonized using 3D morphological thinning to extract its centerline representation.
- 3D Point Ordering: Skeleton points were spatially ordered using a nearest-neighbor approach with orientation constraints to prevent backtracking, ensuring anatomically consistent point sequences along the annulus perimeter.
- Plane Fitting: Principal Component Analysis (PCA) was applied to the ordered annulus points to determine the best-fitting plane, with the plane normal defined as the eigenvector corresponding to the smallest eigenvalue.
- Area Calculation: All segmented structures (leaflets and annulus) were projected onto this optimal plane, and areas were calculated using pixel-based methods with appropriate spatial calibration.

##### 2.2.3.2 Temporal peak detection

Mid-diastole was identified as the frame exhibiting maximum effective valve area, corresponding to the point of maximum valve opening during the cardiac cycle.

#### 2.2.4 Geometric analysis and area quantification

##### 2.2.4.1 Valve plane projection

The projection process involved: I. determination of the optimal valve plane using PCA analysis of annulus centerline points, II. orthogonal projection of all segmented voxels onto this plane, III. conversion to 2D coordinates using orthonormal basis vectors derived from the plane normal, and IV. creation of high-resolution 2D images with pixel sizes calculated from the original voxel spacing and projection angle.

##### 2.2.4.2 Effective orifice area

Functional valve opening area was determined through morphological analysis of the projected segmentation, using flood-fill algorithms to identify the central opening region.

##### 2.2.4.3 Spatial calibration

All measurements were performed in physical units (mm^2^) using voxel spacing information extracted from DICOM headers. The projection method accounted for oblique viewing angles by adjusting pixel sizes based on the angle between the valve plane and the image coordinate system.

#### 2.2.5 Data selection process

4D TEE volumes from both M-TEER (Mitraclip) and surgical MVR patients were included. Only TEE exams with available 3D acquisition, performed at the Montreal Heart Institute starting from 1 March 2024, were used, as raw data extraction was only enabled at the end of February 2024. Each TEE examination was assigned an internal code corresponding to its specific exam type in the institutional database. Only TEE exams performed within 12 months before the M-TEER or MVR were used, provided that the physician’s clinical report with mitral valve analysis and MVA measurement, as performed by a cardiologist, was available. Intraprocedural TEE exams and exams from patients with prior MV procedures were excluded.

#### 2.2.6 Data extraction process

4D TEE volumes meeting the selection criteria were identified through a series of internal SQL scripts executed across complementary databases, including a report database and an exam type database. The identified 4D TEE DICOMs were then transferred to an internal research server using pydicom-batch (https://github.com/MHI-AI-CoreLab/pydicom-batch).

#### 2.2.7 Data cleaning process

An expert cardiologist performed a manual curation process to identify TEE exams in which the mitral valve was acquired and deemed suitable for analysis.

### 2.3 2D-TEE-based automatic MV segmentation: scallop-level analysis

**FIGURE 3:**
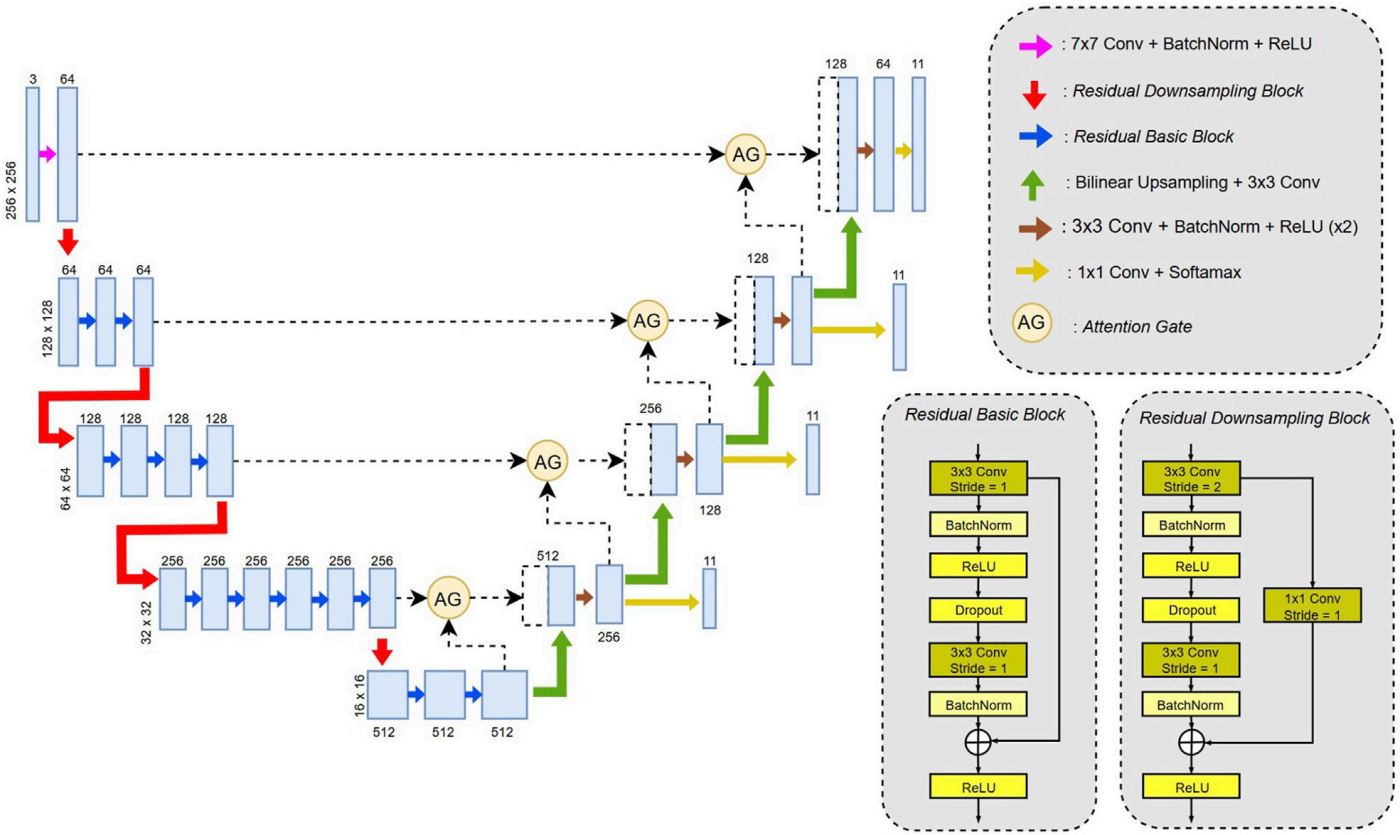
U-Net modified architecture model used for the segmentation of mitral valve leaflets scallops.

For this part, we chose a U-Net architecture (Ronneberger et al., 2015), which is a fully convolutional network consisting of an encoder and a decoder. The model accepts 3-channel ultrasound images x ∈ R3 × 256 × 256 as input and outputs four results: a final segmentation map ϕ(x) ∈ [0,1]11 × 256 × 256 and three deep supervision outputs ψ1(x) ∈ [0,1]11 × 128 × 128, ψ2(x) ∈ [0,1]11 × 64 × 64, and ψ3(x) ∈ [0,1]11 × 32 × 32. Each of the 11 output channels corresponds to one of the following anatomical structures: the six scallops of the mitral valve (A1, A2, A3 for the anterior leaflet, matching P1, P2, P3 respectively for the posterior leaflet), the anterior and posterior papillary muscles, the chordae, the annulus, and the background. We modified the original U-Net architecture to suit our task better, as shown in Figure 3.

#### 2.3.1 Model architecture

##### 2.3.1.1 Encoder

The original encoder has been replaced with a ResNet34-based backbone (Kaiming et al., 2015). Three types of blocks were used: the first is a wide convolution (7 × 7) followed by batch normalization and a ReLU activation function; the second is a residual downsampling block that reduces spatial resolution by a factor of 2 using a 3 × 3 convolution with stride 2; the third is a standard residual block with 3 × 3 convolutions. Both residual block types use skip connections to improve gradient flow during training.

##### 2.3.1.2 Decoder

In the decoder, bilinear up-sampling was used instead of transposed convolutions to reduce checkerboard artifacts (Odena et al., 2016). The rest of the decoder followed the original U-Net structure, with skip connections passed through attention gates, concatenated, and followed by two 3 × 3 convolutions, batch normalization, and ReLU. Each decoder stage also includes a final 1 × 1 convolution and a SoftMax activation to produce intermediate outputs for deep supervision.

##### 2.3.1.3 Attention gates

To improve the focus on the mitral valve and reduce the segmentation of non-relevant muscular structures, we integrated attention gates. These modules, introduced by Oktay et al. (Schlemper et al., 2019), highlight specific regions of interest during training. Each attention gate takes as input a skip connection *x* from the encoder and a gating signal *g* from the corresponding decoder stage and returns a refined feature map with the same dimensionality as *x* (Supplementary Figure 1).

#### 2.3.2 Loss functions

To optimize the segmentation network, we used a combination of Dice loss and Focal loss, which are well-suited for highly imbalanced multiclass segmentation tasks.

##### 2.3.2.1 Dice loss

The generalized Dice loss is defined as follows:

$$L_{Dice}=1-\frac{1}{\left|C^{\prime }\right|}\sum_{c\in C^{\prime }}\frac{2\sum_{i=1}^{N}p_{i,c}\;g_{i,c}+\varepsilon }{\sum_{i=1}^{N}p_{i,c}+\sum_{i=1}^{N}g_{i,c}+\varepsilon }$$

where *C* ^′^ represents the set of classes present in the image, *N* is the number of pixels in the image, *p*_*i,c*_ is the predicted probability for pixel *i* belonging to class *c*, *g*_*i,c*_ is the corresponding ground truth (one-hot encoded), and *ε* is a small constant to avoid division by zero.

##### 2.3.2.2 Focal loss

To further address class imbalance and focus training on hard-to-classify pixels, we also employed the Focal loss (Lin et al., 2018), defined as:

$$L_{Focal}=-\frac{1}{N}\sum_{i=1}^{N}\sum_{c\in C}{\left(1-p_{i,c}\right)}^{\gamma }\;g_{i,c}\;\log\left(p_{i,c}\right)$$

where *C* denotes the set of all classes (not just those in the image), and *γ* is the focusing parameter (set to 2 in this study) that decreases the relative loss contribution of well-classified pixels.

##### 2.3.2.3 Combined loss

The final training objective for a single output is a simple combination of the two losses:

$$L=\frac{L_{Dice}+L_{Focal}}{2}$$

#### 2.3.3 Loss functions with deep supervision

The final loss is applied not only to the network’s final output but also to intermediate outputs. This deep supervision strategy, introduced in (Lee et al., 2014), encourages lower decoder layers to focus on relevant regions early in the network.

Let *ϕ*(*x*) ∈ [0*,*1]^*C*×*H*×*W*^ be the final output, and {*ψ*_*k*_(*x*)}^3^_*k*=1_ be the three intermediate deep supervision outputs. The total loss is then computed as:

$$L_{total}=L\left(\phi \left(x\right),G\right)+\sum_{k=1}^{3}L\left(\psi_{k}\left(x\right),G^{\left(k\right)}\right)$$

Since the intermediate outputs from the decoder have lower spatial resolution than the input image, the corresponding ground truth masks need to be downsampled to match each output size before calculating the loss.

Let *G* ∈ {0*,*1}^*C*×*H*×*W*^ be the original one-hot encoded ground truth mask. For each deep supervision output *ψ*_*k*_(*x*) ∈ [0*,*1]^*C*×*Hk*×*Wk*^, the ground truth is downsampled using nearest-neighbor interpolation:

$$G^{\left(k\right)}=\text{Downsample}\left(G,H_{k},W_{k}\right)$$

where (*H*_*k*_ *,W*_*k*_) are the height and width of the *k*-th intermediate output. Nearest-neighbor interpolation preserves the discrete class labels, ensuring accurate loss computation for each class. The total loss is then computed by comparing each output *ψ*_*k*_(*x*) to its corresponding downsampled ground truth *G* ^(*k*)^.

#### 2.3.4 Optimization and training

The network was trained with the Adam optimizer, starting with a learning rate of 1 × 10^−4^ and a batch size of 24. To prevent overfitting, a weight decay of 1 × 10^−6^ and dropout with a rate of 0.2 in the encoder layers were used. Data augmentation was extensively employed to boost the diversity of the training set, including random rotations (up to 45°), translations, and scaling (between 0.75 and 1.25). These augmentations were applied during training in real-time to improve the model’s ability to generalize.

#### 2.3.5 Dataset and preprocessing

The dataset included 992 TEE images from 77 different patients who underwent a Mitraclip procedure, focusing on the mitral valve, with 11 segmentation classes representing various anatomical structures, with corresponding labels (Table 1). Only 2D images were analyzed. A total of 2,200 ground truth annotations were made by a physician on the Labelbox platform. The data were split into 80% for training (N = 821) and 20% for validation (N = 171), ensuring that all images from the same patient remained in the same subset to prevent data leakage.

**TABLE 1:** Legend of labels used for mitral valve leaflets scallops and sub-apparatus structures annotations.

Anatomical structure	Label
A1	a_1
A2	a_2
A3	a_3
P1	p_1
P2	p_2
P3	p_3
MV chordae	Chordae
MV annulus	Annulus
AL papillary muscle	Papillary_anterior
PM papillary muscle	Papillary_posterior

AL, anterolateral; MV, mitral valve; PM, posteromedial.

Before training, all images were normalized to have zero mean and unit variance. Both images and their corresponding masks were resized to 256 × 256 pixels when needed.

#### 2.3.6 Evaluation

The segmentation performance was assessed using multiple metrics, including Dice coefficient, precision, recall, and false positive rate (Supplementary Figure 2).

The Dice score is a widely used metric to assess segmentation performance by quantifying the spatial overlap between the predicted segmentation and the ground truth. It is defined as:

$$\text{Dice}=\frac{2\left|A\cap B\right|}{\left|A\right|+\left|B\right|}$$

where *A* represents the predicted segmentation and *B* the reference segmentation.

A Dice score of 1 indicates perfect agreement, whereas a score of 0 indicates no overlap.

This metric is particularly well-suited for medical image analysis because it remains robust to class imbalance (e.g., small anatomical structures occupying only a fraction of the image) and has become a standard benchmark for evaluating segmentation algorithms.

All experiments were conducted in PyTorch 2.6 and trained on an NVIDIA RTX A600 GPU.

## 3 Results

### 3.1 Automatic classification of 2D- TTE and TEE images: quality views assessment

**FIGURE 4:**
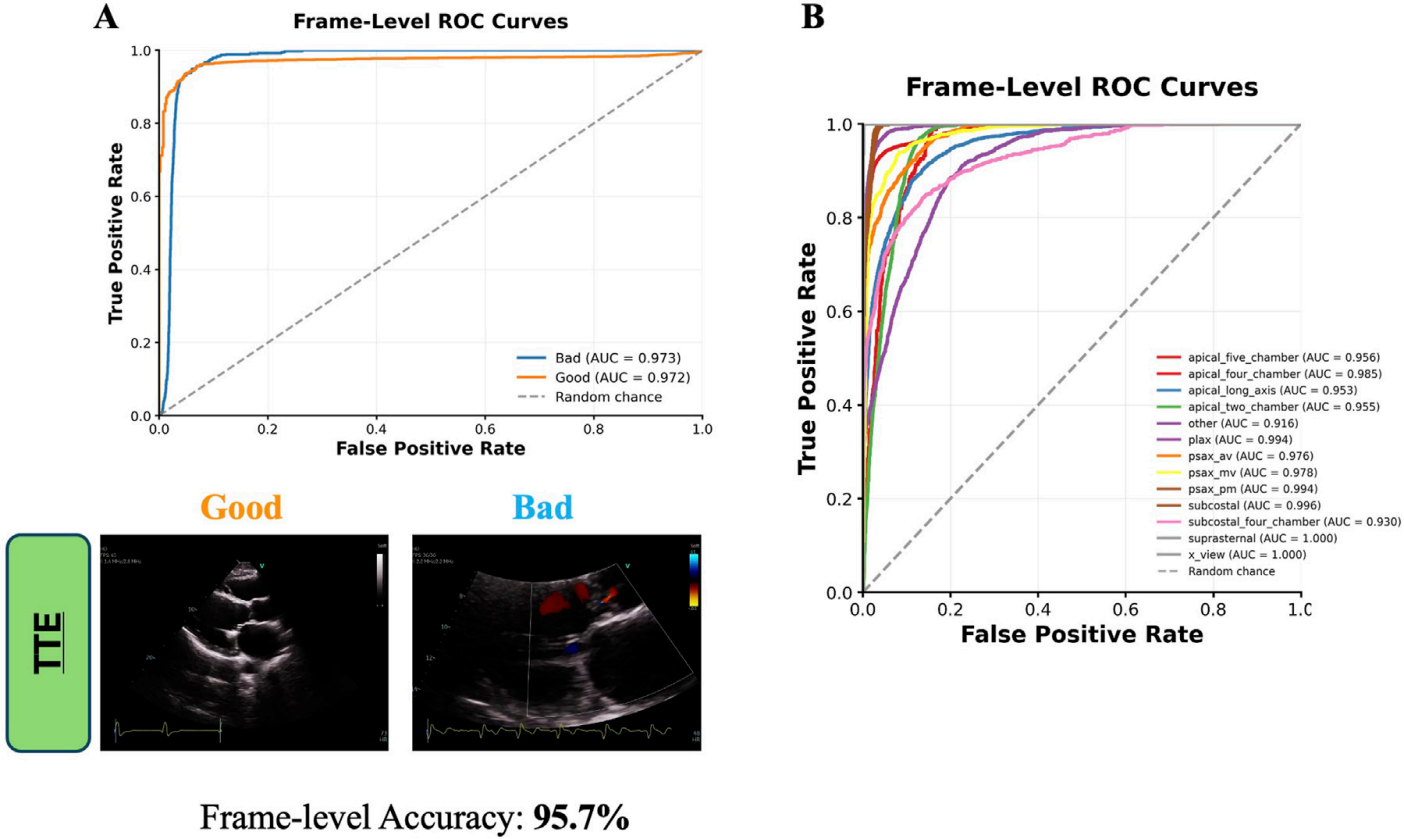
Frame-Level ROC curves for TTE Quality Image assessment **(A)** and Views classification **(B)**. TTE images are direct outputs from the algorithm to illustrate the classification of “good” versus “bad” views. AUC = area under curve; av = aortic valve; mv = mitral valve; pm = papillary muscle; TTE = transthoracic echocardiography; plax = parasternal long axis; psax = parasternal short axis; ROC = receiver operating characteristic curve.

**FIGURE 5:**
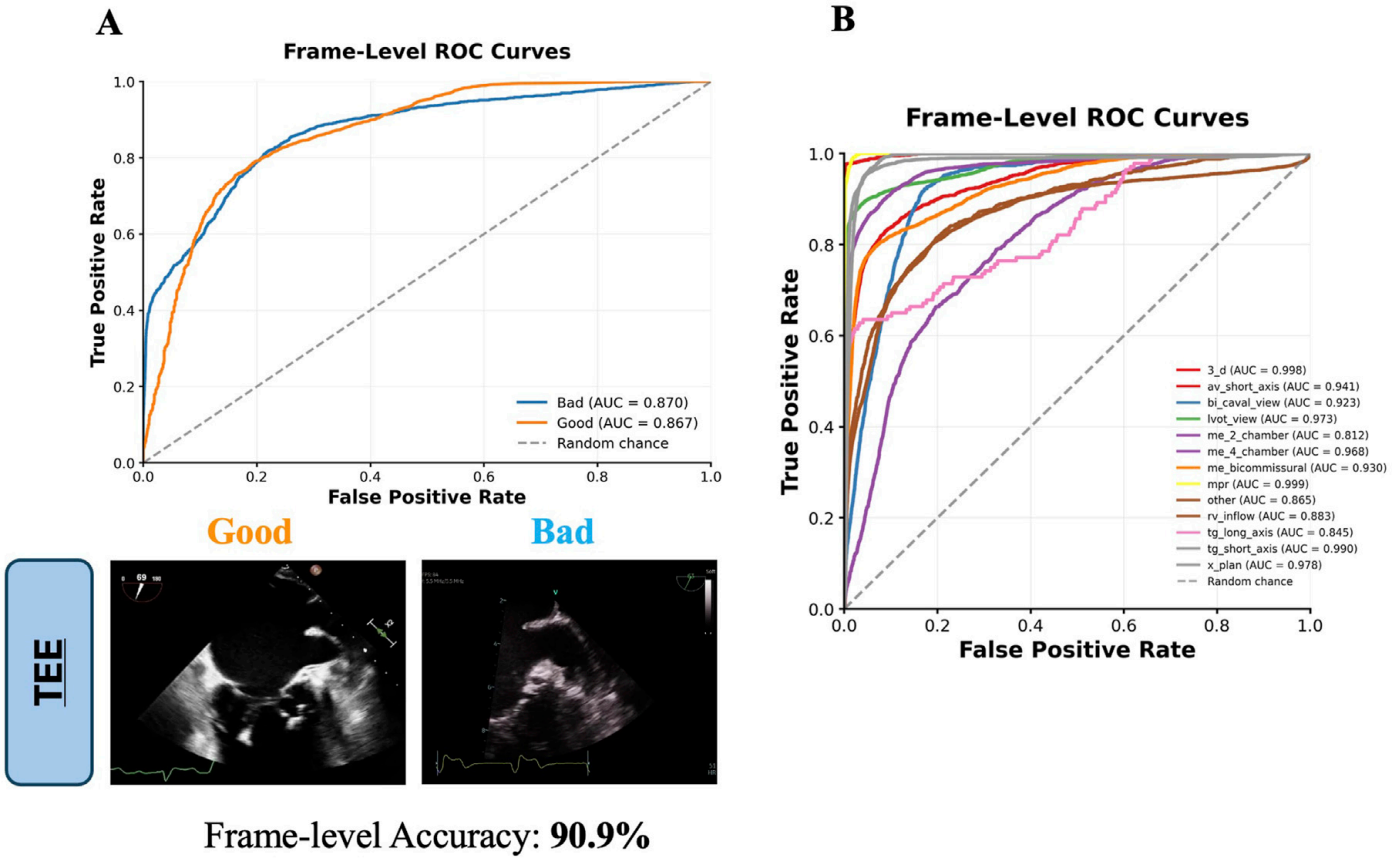
Frame-Level ROC curves for TEE Quality Image assessment **(A)** and Views classification **(B)**. TEE images are direct outputs from the algorithm to illustrate the classification of “good” versus “bad” views. AUC = area under curve; av = aortic valve; me = mid-esophageal; mpr = multiplanar reconstruction; rv = right ventricle; tg = transgastric; lvot = left ventricular outflow tract; 3_d = three-dimensional; TEE = transesophageal echocardiography; ROC = receiver operating characteristic curve.

ECHO-PREP first algorithm was trained to identify key diagnostic views in TTE and diagnostic and procedural views in TEE examinations (algorithm 1, Figure 1) to determine the level of image quality needed for an M-TEER, based on a dataset of 530 TTE and 800 TEE pre-M-TEER acquisitions, respectively. The total number of TTE and TEE analyzed frames was 58.749 and 52.058, respectively. The dataset distribution of TTE and TEE diagnostic views is shown in Supplementary Tables 1, 2, respectively. The algorithm successfully determined whether the TTE was of good quality with a frame-level accuracy of 95.7% (Figure 4A) and performed well in view classification (Figure 4B). For TEE, the algorithm produced similar results, accurately identifying whether TEE views were of sufficient quality for patient eligibility and procedural guidance of M-TEER in 91% of cases, with a high overall accuracy for TEE view classifications, as demonstrated by AUC values (Figure 5).

### 3.2 4D-TEE-based automatic MV segmentation and MV area measurements

**FIGURE 6:**
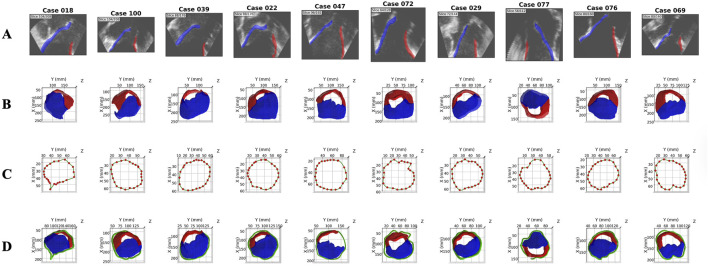
Enhanced Annotation from MVSEG 2023: 10 representative cases. Rows **(A)** 2D sagittal slice showing original echocardiography with leaflet segmentation overlay. **(B)** 3D superior view of MVSEG 2023 baseline dataset (leaflets only). **(C)** Manual annulus annotation with control points and B-spline fitting. **(D)** Final enhanced dataset with complete mitral valve (leaflets + annulus). 2D = two-dimensional; 3D = three-dimensional.

**FIGURE 7:**
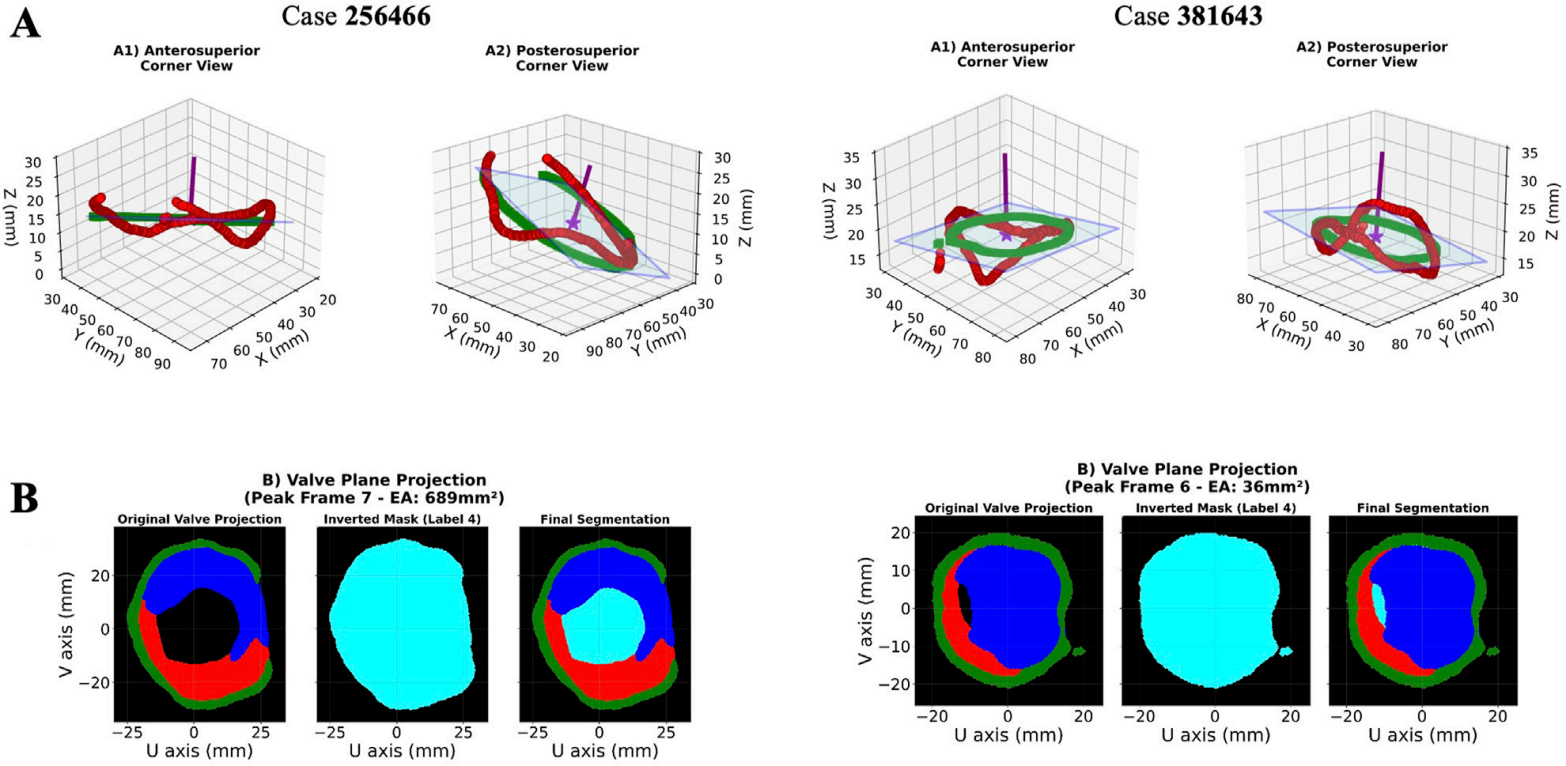
PCA-Based Optimal Plane Projection for 3D Mitral Valve Quantification: 2 representative cases. Rows **(A)** 3D visualization of annulus skeleton points (red) and their projection onto the PCA-derived optimal plane (green squares) from anterosuperior (A1) and posterosuperior (A2) viewpoints. The semi-transparent blue plane represents the best-fitting 2D projection surface with a normal vector (purple). **(B)** Valve plane projection showing effective area measurement from the peak cardiac frame with color-coded anatomical structures (posterior leaflet = red, anterior leaflet = blue, annulus = green, functional area = light blue). 2D = two-dimensional; 3D = three-dimensional; PCA = Principal component analysis.

**FIGURE 8:**
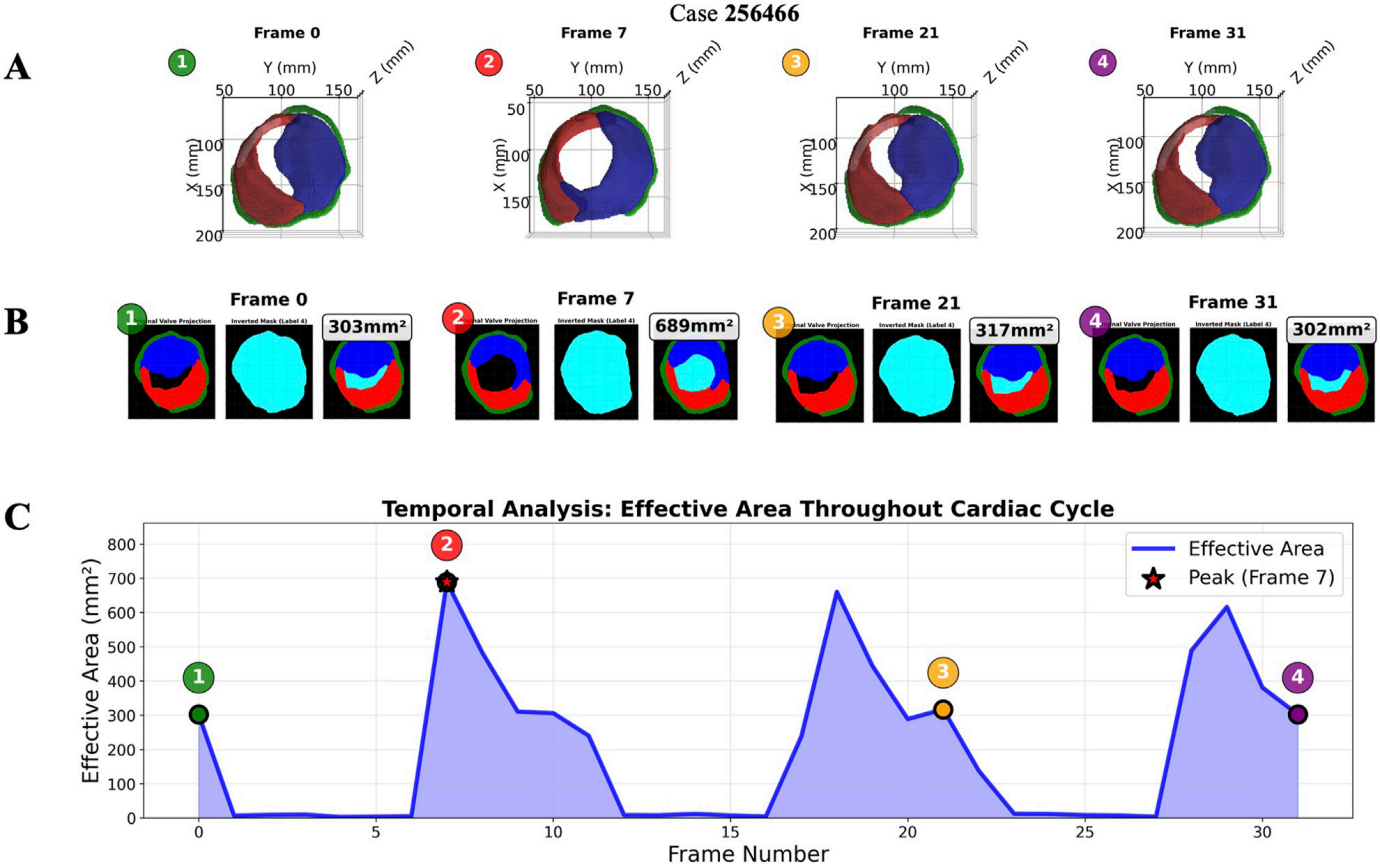
Temporal analysis showing valve geometry evolution across the cardiac cycle: representative case. Rows **(A)** 3D-views and **(B)** 2D-projections at four time points (posterior leaflet = red, anterior leaflet = blue, annulus = green, functional area = light blue). **(C)** Effective area curve with peak detection (red star) and frame markers (numbered circles). 2D = two-dimensional; 3D = three-dimensional.

ECHO-PREP second algorithm was trained on a total of 135 TEE 4D volumes from the MVSEG2023 dataset, with a 70%–30% split for training and validation, respectively. Segmentation of relevant anatomical features, including the mitral anterior and posterior leaflets and annulus, was performed using the MONAI Auto3DSeg software after identifying the mid-diastole frame (Figure 6). A logical stepwise understanding from anatomy to segmentation can be derived from Figure 7, which effectively illustrates the use of a PCA-based optimal plane and segmentation pipeline for valve analysis. The figure clearly contrasts two cases (256466 vs. 381643) using 3D visualizations (on top) and 2D valve plane projections (below) at the peak frame. In Case 256466 (left panel), the effective area (EA) is much larger (689 mm^2^), and the valve appears more symmetric and complete in both 3D and projection views. Segmentation appears clean, with well-demarcated leaflets. In case 381643 (right panel), the EA is significantly smaller (36 mm^2^), indicating severe restriction. The 3D views show distorted, irregular geometry, and the projection and segmentation reveal significant leaflet malcoaptation or incomplete opening. Combining 3D multi-angle views with 2D projection and segmentation provides complementary perspectives: the segmentation masks (original, inverted, final) offer transparency into the algorithm’s steps and show good alignment between the quantitative data (EA values) and visual impression. The contrast with case 256466 (EA 689 mm^2^) highlights the method’s robustness in capturing extreme phenotypes. The added value of a comprehensive temporal analysis of mitral valve dynamics is demonstrated in Figure 8. Instead of static geometry, it captures the valve’s physiological motion and functional variability. The top row shows 3D superior views at four timepoints across the cardiac cycle (start-peak-mid-end), with valve structures clearly delineated. It demonstrates valve opening dynamics, from partial opening at Frame 0 to maximal separation at Frame 7. The middle row shows 2D valve projections at the same key frames. Effective orifice area (EA) values are: start: 303 mm^2^, peak: 689 mm^2^, mid: 317 mm^2^, end: 302 mm^2^. This visualization complements the 3D view by quantifying leaflet separation. The bottom panel displays the temporal analysis graph with EA plotted across all 32 frames: peak EA occurs at Frame 7 (689 mm^2^). The cycle demonstrates typical dynamic variation, with large fluctuations between systolic closure and diastolic opening (mean EA: 198 mm^2^; range: 3–689 mm^2^). This patient (case 256466) shows normal dynamic opening and closure patterns, with a large peak EA, consistent with preserved mitral valve function. The data highlights the algorithm’s ability to continuously track valve dynamics throughout the cardiac cycle, not just at isolated frames. The temporal profile offers a clear functional fingerprint that could distinguish healthy from pathological valves.

**FIGURE 9:**
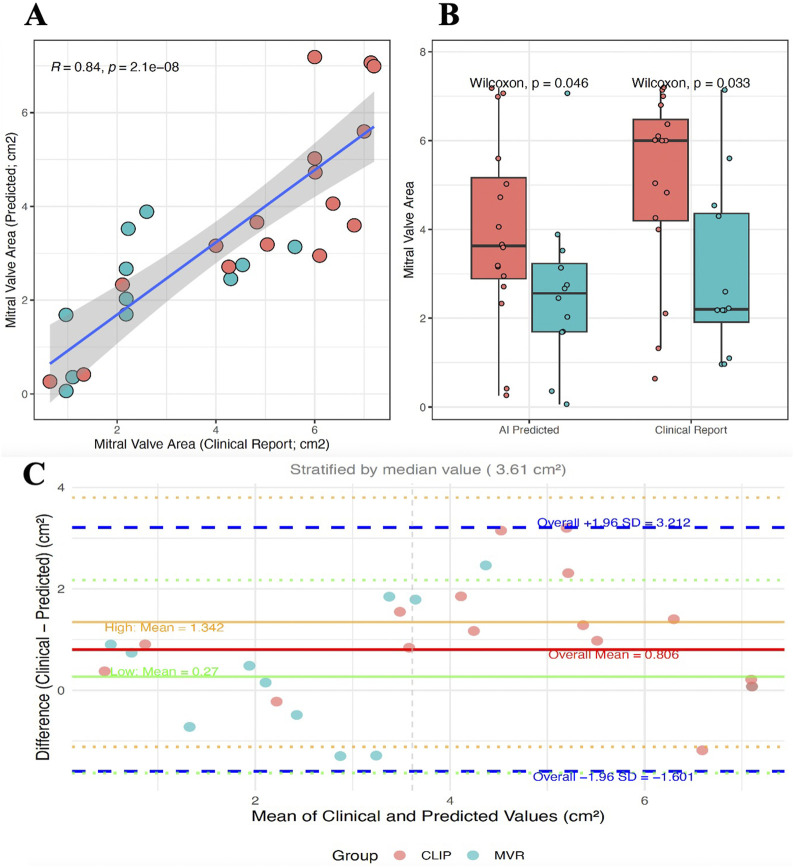
Validation of AI-Based Mitral Valve Area Quantification Against Clinical Reports. **(A)** Correlation between clinical reports and AI predictions showing strong agreement (R = 0.84). **(B)** Distribution comparison between measurement methods for Mitraclip (red) and surgical MVR (blue) patient groups. Wilcoxon tests show statistical significance of group differences. **(C)** Bland–Altman plot assessing agreement between clinical and AI-predicted mitral valve area, stratified by median value (3.61 cm^2^). The overall mean difference was 0.806 cm^2^ (solid red line). Agreement was better in the low-value group (mean difference 0.27 cm^2^, solid green line) compared to the high-value group (mean difference 1.34 cm^2^, solid orange line). Dashed blue lines indicate the 95% limits of agreement (mean ± 1.96 SD). MVR = mitral valve replacement; SD = standard deviation.

The validation of the algorithm for quantifying the mitral EA involved analyzing a total of 221 TEE 4D volumes performed at our center as part of a pre-procedural assessment of mitral regurgitation. Images were divided into two groups: those from patients who later underwent M-TEER with Mitraclip (121 4D volumes from 30 patients) and those from patients who had surgical mitral valve replacement (100 4D volumes from 18 patients). A physician reviewed the available images from the center’s database and preliminarily excluded videos with unsuitable views for calculating the MVA, such as poor image quality, artifacts, or the presence of a previous surgical prosthesis or valve ring. The validation of the AI-predicted MVA quantification was performed by comparing it to the gold standard of manual measurements from physician clinical reports. In Figure 9A, the scatter plot shows a strong positive correlation (Pearson’s R = 0.84) between the MVA measurements from clinical reports and those predicted by our algorithm. The correlation is statistically significant (p < 0.001), demonstrating excellent agreement between the AI and human expert measurements.

Further validation was conducted through group discrimination. Since our center handles high volumes and specializes in mitral repair with a high success rate across various mitral regurgitation scenarios, it was assumed that patients who ultimately underwent surgical MVR were more likely to have a non-repairable valve due to factors like a stenotic or restrictive valve with a smaller MVA, after excluding patients with endocarditis or prior valve procedures. In Figure 9B, the box plots compare the distribution of MVA measurements between two patient groups: Mitraclip (red) and surgical MVR (blue). As expected, the MVR group exhibits a significantly smaller mitral valve area. The difference between the two groups is statistically significant for both measurement methods (clinical reports: Wilcoxon p = 0.033; AI predictions: Wilcoxon p = 0.046). A Bland-Altman analysis was performed to complement the correlation and illustrate agreement between AI-derived and physician-reported mitral valve area (Figure 9C)*.*

Even with some limitations, this remains an important validation step, showing that the algorithm not only aligns with clinical reports on individual measurements but also keeps the clinically relevant physiological differences between different patient groups. The higher significance (p = 0.033) in the clinical reports is expected, as they are the reference standard.

### 3.3 2D-TEE-based automatic MV segmentation: scallop-level analysis

**FIGURE 10:**
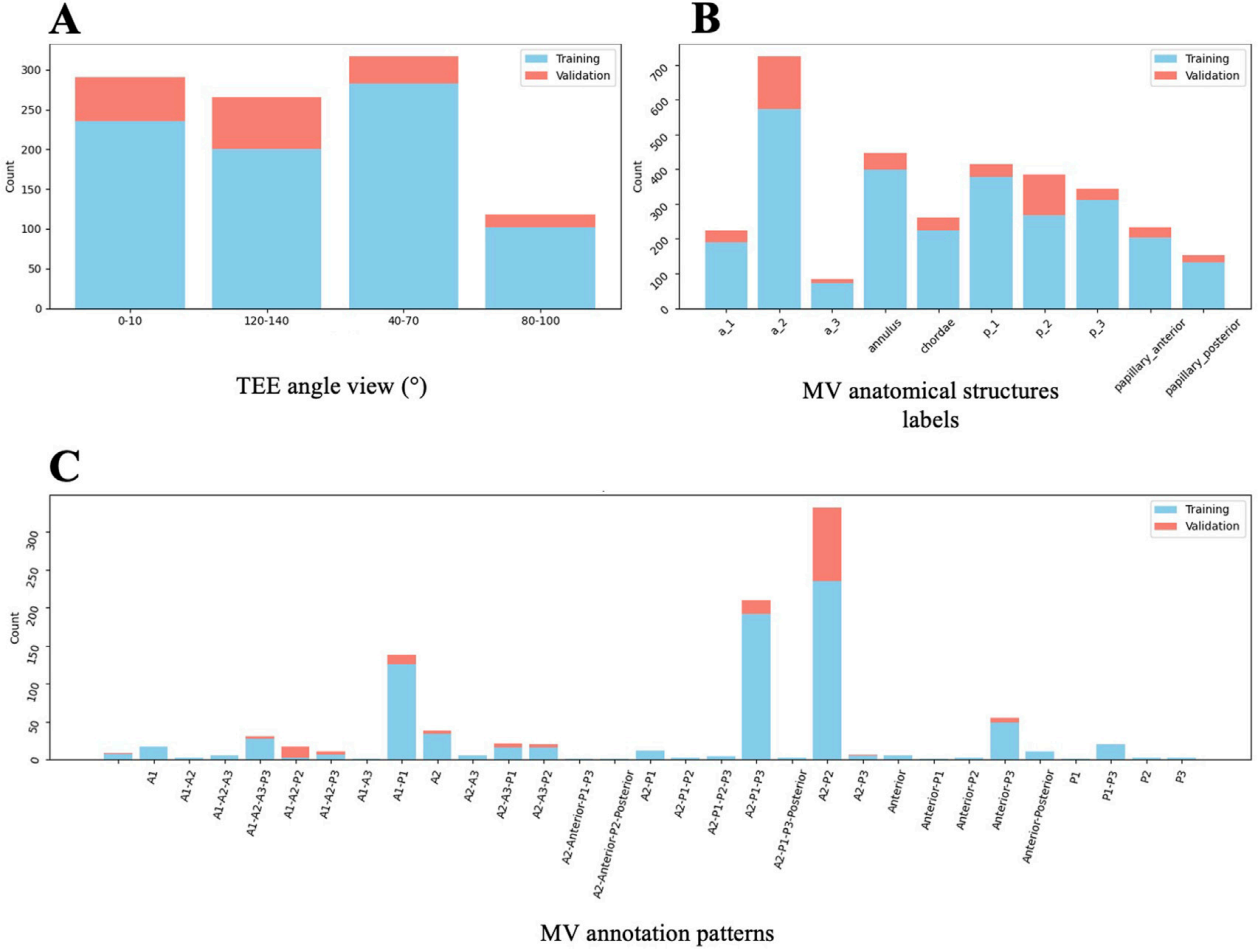
Dataset overview for 2D TEE Segmentation Model (Algorithm 3). Distribution of TEE angle views **(A)**, anatomical MV structures **(B)**, and MV annotation patterns **(C)** from the analysed image dataset, divided into training and validation subsets. 2D = two-dimensional; MV = mitral valve; TEE = transoesophageal echocardiography.

**FIGURE 11:**
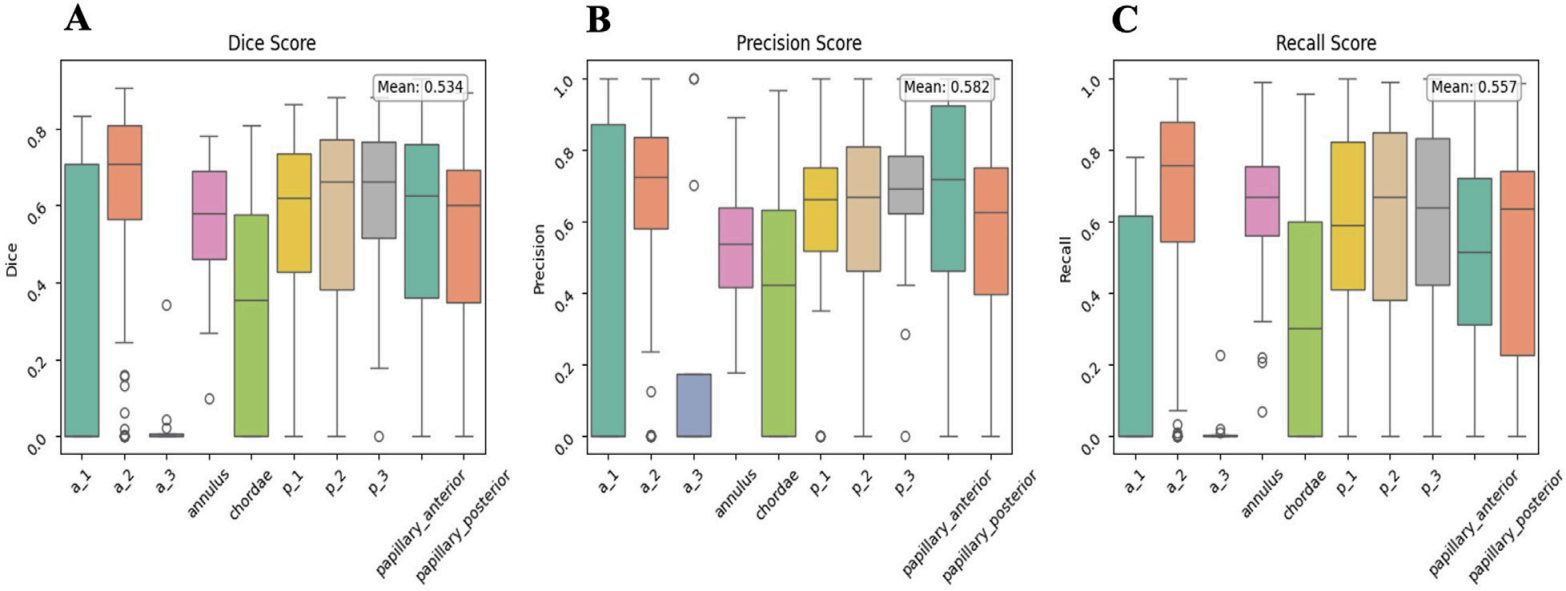
Global validation results for the 2D TEE Segmentation Model (Algorithm n.3). Mean Dice **(A)**, Precision **(B)**, and Recall **(C)** scores are displayed for all MV labels. Annotation labels correspond to the respective MV structures as previously defined. 2D = two-dimensional; MV = mitral valve; TEE = transoesophageal echocardiography.

A dataset overview, showing the distribution of TEE angle views and annotations of the MV structures during segmentation, is presented in Figure 10. The most common anatomical patterns were A1-P1, A2-P2, and A2-P1-P3 (Figure 10C). These were mainly mid- esophageal (ME) five- (5C) and four-chamber (4C) views, ME long axis, and ME commissural views, primarily used to evaluate the MV, especially in pre-procedural M-TEER assessment. The overall validation results are shown in Figure 11, with a mean Dice score of 0.534 across the entire validation dataset. Individual Dice scores for each MV structure annotation are listed in Table 2. As expected, performance is slightly lower in commissural regions such as A3 and A1. When analyzing these results, it is essential to note that if the segmentation involves a small anatomical structure, such as a short or retracted posterior mitral leaflet, which is common in functional mitral regurgitation, the metric results, particularly the Dice score, may be misleading.

**TABLE 2:** Segmentation performance metrics for mitral valve structures annotations, as identified with their labels.

Label	Dice	Precision	Recall	FPR
a_1	0.289 ± 0.352	0.369 ± 0.432	0.253 ± 0.318	0.000 ± 0.001
a_2	0.640 ± 0.236	0.673 ± 0.233	0.671 ± 0.274	0.001 ± 0.001
a_3	0.034 ± 0.098	0.225 ± 0.414	0.021 ± 0.065	0.000 ± 0.000
Annulus	0.557 ± 0.162	0.520 ± 0.168	0.639 ± 0.193	0.002 ± 0.001
Chordae	0.342 ± 0.292	0.393 ± 0.313	0.344 ± 0.319	0.001 ± 0.001
p_1	0.548 ± 0.266	0.599 ± 0.272	0.566 ± 0.310	0.001 ± 0.001
p_2	0.550 ± 0.281	0.605 ± 0.287	0.583 ± 0.324	0.001 ± 0.001
p_3	0.607 ± 0.205	0.682 ± 0.186	0.609 ± 0.272	0.000 ± 0.001
Papillary anterior	0.546 ± 0.247	0.647 ± 0.295	0.526 ± 0.269	0.002 ± 0.002
Papillary posterior	0.492 ± 0.297	0.537 ± 0.329	0.514 ± 0.333	0.002 ± 0.003

Values are Mean ± Standard Deviation.

FPR, false positive risk.

**FIGURE 12:**
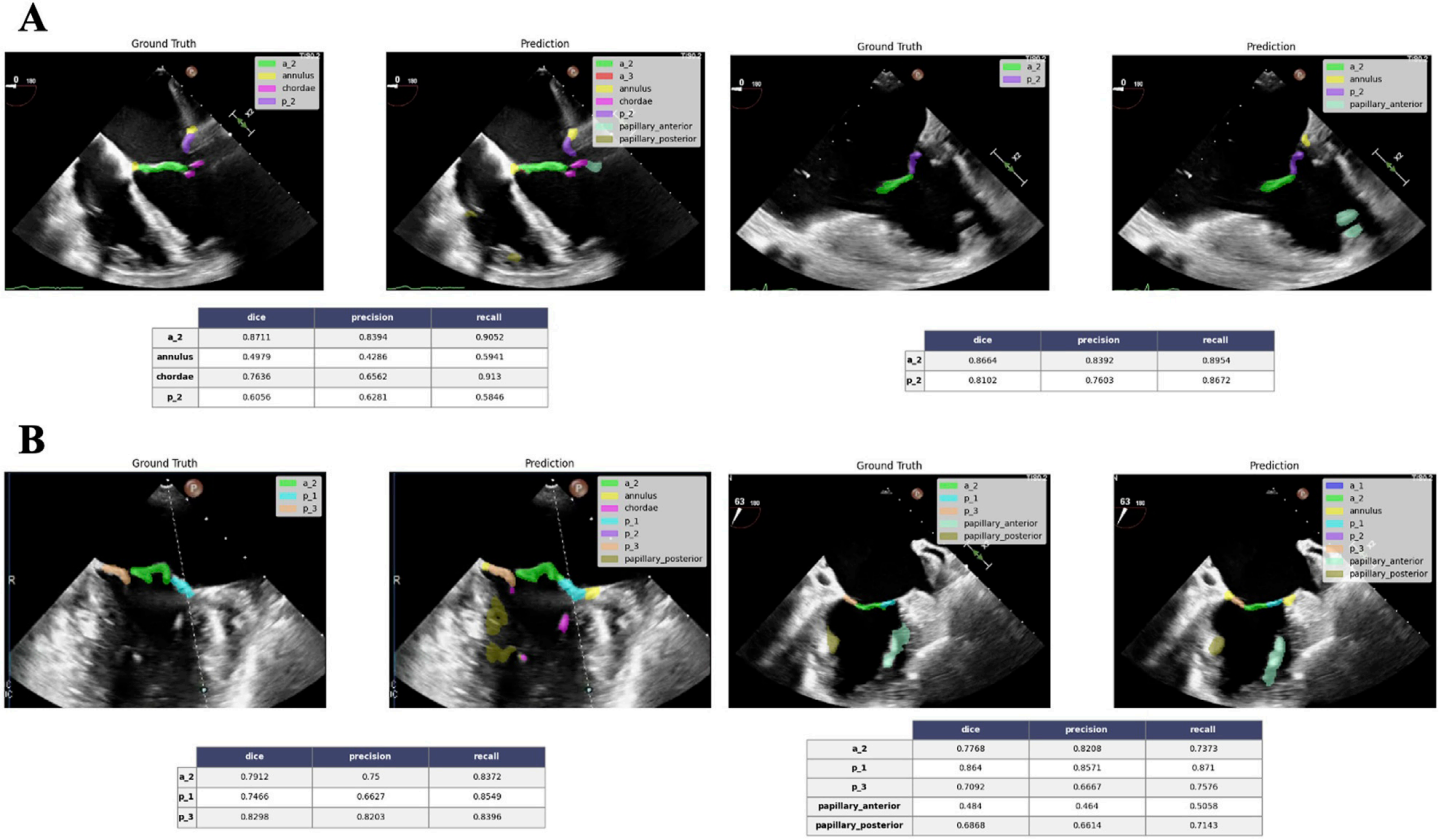
Good performance segmentation example. ME 4C views at 0° **(A)** and ME commissural views at 63° **(B)** show good correlation between the ground truth and the neural network predicted annotation for A2-P2 and P3-A2-P1 combinations, respectively. Tables (below) show the corresponding performance metrics score for each label annotation. ME = mid-esophageal.

Generally, a Dice score above 0.7 is considered a good (visually) result. Regarding leaflet scallop segmentation, the ME Long Axis views and ME Commissural views are typically segmented very well by the neural network, with the P3-A2-P1 sequences being highly represented in the dataset. An example is illustrated in Figure 12. However, the results are less accurate for other views. The poorer outcomes mainly stem from the ME 4 C and ME 5 C views, which often confuse the A2- P2 and A1- P1 sequences. The segmentation of the annulus produces quite good results. It is important to note that in 2D images, annulus annotation is very small and can be biased when calcifications are absent, making the Dice score very sensitive. Even in images without annulus annotations, the neural network seems capable of detecting the annulus correctly. Since the papillary muscles and mitral chordae are located within the ventricle (mostly represented by dark pixels), the network is highly sensitive to noise and bright areas within the ventricle, which can lead to confusion with these structures. As a result, the outcomes for the papillary muscles- and even more so for the chordae- are not optimal.

## 4 Discussion

In this study, we present ECHO-PREP, an integrated multi-stage deep learning framework for pre-procedural mitral valve assessment in candidates for M-TEER procedure. Our approach encompasses three complementary modules: automated quality assessment of echocardiographic views, 4D segmentation with functional valve area quantification, and 2D scallop-level analysis of valve anatomy. Together, these components aim to address the current challenges of variability, subjectivity, and inefficiency in echocardiographic interpretation for M-TEER planning.

The first significant finding was the high performance of the quality assessment algorithm for both TTE and TEE images, achieving frame-level accuracies above 90%. This step, although often overlooked, is clinically critical: poor-quality imaging is a common reason for inconclusive evaluations and may delay intervention. By introducing automation at this stage, our framework could improve workflow efficiency and ensure that downstream analyses are only performed on diagnostically valid inputs. The second major result was the successful implementation of 4D TEE-based segmentation with automated mitral valve area (MVA) quantification. The algorithm showed strong correlation with physicians’ clinical reports (R = 0.84, p < 0.001), confirming its reliability for valve sizing and functional assessment. Notably, the system not only reproduced static area measurements but also captured temporal variations of valve opening and closure, offering a dynamic fingerprint of valve physiology. This longitudinal perspective may become a powerful discriminator between repairable and non-repairable valves, as suggested by the observed differences between patients undergoing M-TEER and those treated with surgical valve replacement.

However, the validation of MVA quantification across patient groups relied on the assumption that all surgically replaced valves (MVR) were non-repairable. In our high-volume center, which has a strong track record of surgical valve repair, it is reasonable to infer that patients selected for MVR likely presented with severely remodeled or rheumatic valves, resulting in significantly smaller valve areas in this group. Nevertheless, even after applying strict exclusion criteria, additional factors may have influenced the decision to replace rather than repair, thereby weakening the correlation between smaller MVA and surgical replacement. This limitation reduces the ability of the AI model to correctly classify MVR patients based solely on pre-procedural imaging. Moreover, the retrospective design and the single-center context limit the generalizability of our findings, particularly in centers with lower surgical expertise in mitral valve repair.

Finally, the scallop-level analysis represents a novel and ambitious contribution toward standardized, automated scallop identification, a task that today relies heavily on operator expertise. While segmentation of large structures (annulus, anterior and posterior leaflets, central scallops) reached acceptable accuracy, finer anatomical elements such as commissural leaflet scallops or chordae tendinae were more difficult to identify consistently. The mean Dice score of 0.53 on the overall structures dataset reflects these challenges. Nonetheless, the network correctly reproduced frequent anatomical configurations (e.g., A2-P2, P1-A2-P3), especially in mid-esophageal long-axis and commissural views, which are crucial for procedural planning. This constitutes a meaningful step. One important consideration in interpreting scallop-level results is the potential role of overfitting. Our dataset, while curated and enriched with physician annotations, remains limited in size compared to the complexity of the task. Neural networks trained on relatively small, homogeneous datasets are prone to overfitting, i.e., capturing dataset-specific patterns rather than generalizable features. This phenomenon may explain why performance was higher in anatomical regions and views more frequently represented in the training set (e.g., A2-P2 in long-axis views), while less common configurations showed reduced accuracy. Overfitting risk is further heightened by the high class imbalance inherent in scallop annotation: commissural scallops, papillary muscles, and chordae are both smaller in size and underrepresented, leading to disproportionate errors in Dice score evaluation. Another factor to consider is that Dice scores, while informative, may not fully reflect clinical usability. For small structures, a low Dice value may correspond to visually acceptable segmentation. Conversely, a higher Dice in a large structure might still fail to capture clinically relevant details such as leaflet clefts or tethering. This highlights the need for evaluation metrics that combine geometric accuracy with clinical relevance, possibly integrating expert qualitative scoring.

Compared to earlier approaches, which focused on annulus-only segmentation or static 3D models (Costa et al., 2019; Carnahan et al., 2021; Aly et al., 2022; Chen et al., 2023; Munafò et al., 2024; Andreassen et al., 2019; Andreassen et al., 2022), our pipeline integrates quality control, 4D functional analysis, and scallop-level anatomy into a unified framework. Recent semi-supervised methods (Munafò et al., 2025) demonstrated reliable 4D segmentation, but they did not extend to scallop analysis or clinical validation against surgical and percutaneous cohorts.

By validating our algorithm against both manual measurements and group-level clinical outcomes, we provide an important translational step toward clinical applicability.

### 4.1 Limitations and core challenges

Several limitations should be taken into account. Our datasets are relatively modest and partially monocentric, raising concerns about generalizability. Although validation against clinical reports is encouraging, clinical measurements and annotations themselves are subject to intra- and inter-operator variability, especially for complex structures like scallops and pathology zones, which could influence correlations. This is the concept of the noisy ground truth: an AI model trained on one expert’s labels may perform poorly when judged by another expert’s standards, highlighting the inherent ambiguity in the task. Scallop-level segmentation performance is limited and susceptible to overfitting and to the “rare event” challenge: pathologies like commissural lesions, complex Barlow’s disease with multiple prolapses, or specific calcification patterns are less frequent than standard A2/P2 pathologies. A deep learning model trained on an imbalanced dataset will inherently be biased towards the more common cases and will struggle with these rare but clinically crucial edge cases. External validation on larger, more diverse datasets will be essential to confirm robustness.

Finally, while our system successfully quantifies pre-procedural imaging, its real-time intra-procedural utility remains untested. A model can perfectly segment a valve and measure lengths, but determining the feasibility and the clip strategy requires synthesizing all that information into a clinical decision. This involves tacit knowledge that cardiologists and cardiac surgeons accumulate over years and that is rarely explicitly stated in the annotations (e.g., “leaflet is too fragile,” “coaptation gap is too wide for a single clip,” “the jet is too commissural for a safe grasp”).

### 4.2 Future perspectives

Moving forward, expanding annotated datasets, ideally through multi-center collaborations and semi-automated labeling strategies, will be crucial to mitigate overfitting and improve generalizability. Incorporating advanced architectures (e.g., vision transformers or hybrid CNN–transformer models) and uncertainty quantification methods may further enhance reliability in challenging cases. Using the STAPLE algorithm (Simultaneous Truth and Performance Level Estimation) or similar statistical methods to generate a probabilistic “consensus truth” from multiple annotations could also help in building a more consolidated dataset for training. The use of generative AI techniques like Generative Adversarial Networks (GANs) or diffusion models could help to create realistic synthetic examples of rare and challenging cases to balance the training set. Furthermore, the segmentation of the valve informs the pathology classification, which tells the feasibility prediction. Design a single model that simultaneously learns to segment, classify views, classify pathology, and detect calcifications makes the model more robust and generalizable than a set of separate models.

Dabiri et al. (2022) conducted a simulation study to assess how the number and location of MitraClips influence residual MR and valve hemodynamics. This study emphasizes that procedural success depends not only on patient selection but also on real-time strategic decisions regarding clip quantity and positioning.

In fact, beyond pre-procedural planning, a promising future direction involves integrating DL into intra-procedural guidance. Real-time segmentation and scallop identification could assist operators during clip placement by continuously updating valve anatomy and coaptation maps as the device interacts with the leaflets. Automated tracking of leaflet grasping zones and prediction of residual regurgitation jets could help reduce procedure time, cut down on unnecessary clip deployments, and improve procedural safety. Such integration would need further optimization of inference speed, user-friendly visualization tools, and compatibility with procedural echocardiography systems. Ultimately, combining imaging-derived AI quantification with biomechanical simulation could create a comprehensive decision-support system, predicting both procedural feasibility and the hemodynamic trade-offs of various clip strategies.

The most transformative future direction, however, involves a core shift from a reconstructive to a predictive and simulative model. Current models, including our own, analyze the pre-procedural anatomy in a static way. One future use of our ECHO-PREP workflow will be to train the model on paired data: pre-procedural 3D TEE volumes and their corresponding post-procedural 3D TEE volumes with the clip deployed and a good result. Instead of just identifying what exists, the AI will learn what a successful outcome looks like and apply that knowledge to guide the pre-procedural plan, by understanding the mechanical changes caused by clip implantation on the valve and the optimal morphological features of a pre-procedural valve that lead to a successful post-procedural result. This “backward-forward” AI approach has the highest potential to truly standardize and democratize M-TEER planning worldwide, allowing less experienced centers to leverage the collective expertise embedded in the AI from high-volume centers, all while using the standard imaging equipment they already have. These analyses, together with the integration of fluid-dynamics simulations, are envisioned as central elements of a comprehensive multi-imaging simulation platform for transcatheter procedures, which we are currently advancing through our ongoing multicenter study, ENVISAGE (NCT07213531).

If validated prospectively, this capability could transform ECHO-PREP from a pre-procedural planning tool into a real-time decision-support system integrated into the cath lab workflow. We envision a future where the interventional cardiologist is empowered not just with tools, but with foresight. This is the true promise of AI: not to replace physicians, but to enhance their capabilities, making their expertise more powerful, precise, and accessible to every patient in need.

## Data Availability

The raw data supporting the conclusions of this article will be made available by the authors, without undue reservation.

## Abbreviations

AI, Artificial intelligence; MR, Mitral regurgitation; TTE, Transthoracic Echocardiography; TEE, Transesophageal echocardiography; M-TEER, Mitral transcatheter edge-to-edge repair; DL, Deep learning.

## References

[B1] Aly A. Khandelwal P. Aly A. Kawashima T. Mori K. Saito Y. (2022). Fully automated 3d segmentation and diffeomorphic medial modeling of the left ventricle mitral valve complex in ischemic mitral regurgitation. Med. Image Anal. 80, 102513. 10.1016/j.media.2022.102513 35772323

[B2] Andreassen B. Veronesi F. Gerard O. Solberg A. Samset E. (2019). Mitral annulus segmentation using deep learning in 3-D transesophageal echocardiography. IEEE J. Biomed. Health Infor 24, 994–1003. 10.1109/JBHI.2019.2959430 31831455

[B3] Andreassen B. Völgyes D. Samset E. Solberg A. (2022). Mitral annulus segmentation and anatomical orientation detection in TEE images using periodic 3D CNN. IEEE Access 10, 51472–51486. 10.1109/access.2022.3174059

[B4] Carnahan P. Moore J. Bainbridge D. Eskandari M. Chen E. Peters T. (2021). “DeepMitral: fully automatic 3D echocardiography segmentation for patient specific mitral valve modelling,” in Medical image computing and computer assisted Intervention-MICCAI 2021: 24th international conference, strasbourg, France, September 27-October 1, 2021, proceedings, part V 24, 459–468.

[B5] Chen J. Li H. He G. Yao F. Lai L. Yao J. (2023). Automatic 3D mitral valve leaflet segmentation and validation of quantitative measurement. Biomed. Sig Process Control 79, 104166. 10.1016/j.bspc.2022.104166

[B6] Costa E. Martins N. Sultan M. Veiga D. Ferreira M. Mattos S. (2019). Mitral valve leaflets segmentation in echocardiography using convolutional neural networks. 2019 IEEE 6th Portuguese Meet. Bioeng. (ENBENG), 1–4. 10.1109/enbeng.2019.8692573

[B7] Dabiri Y. Mahadevan V. S. Guccione J. M. Kassab G. S. (2022). A simulation study of the effects of number and location of MitraClips on mitral regurgitation. JACC Adv. 1 (1), 100015. 10.1016/j.jacadv.2022.100015 38939090 PMC11198285

[B8] Elaziz E. A. Al‐qaness M. Dahou M. Alsamhi S. H. Abualigah L. Ibrahim R. A. (2023). Evolution toward intelligent communications: impact of deep learning applications on the future of 6G technology. Wiley Interdiscip. Rev. Data Min. Knowl. Discov. 14, e1521. 10.1002/widm.1521

[B9] Hien M. Großgasteiger M. Weymann A. Rauch H. Rosendal C. (2014). Reproducibility in echocardiographic two-and three-dimensional mitral valve assessment. Echocardiography 31, 311–317. 10.1111/echo.12365 24028385

[B10] Kaiming H. Zhang X. Ren S. Sun J. (2015). Deep residual learning for image recognition. arXiv:1512.03385. Available online at: https://arxiv.org/abs/1512.03385.

[B11] Leclerc S. Smistad E. Pedrosa J. Ostvik A. Cervenansky F. Espinosa F. (2019). Deep learning for segmentation using an open large-scale dataset in 2D echocardiography. IEEE Trans. Med. Imaging 38 (9), 2198–2210. 10.1109/TMI.2019.2900516 30802851

[B12] Lee C. Xie S. Gallagher P. Zhang Z. Tu Z. (2014). Deeply-supervised nets. arXiv:1409.5185. 10.48550/arXiv.1409.5185

[B13] Lin T. Goyal P. Girshick R. He K. Dollár P. (2018). Focal loss for dense object detection. arXiv: 1708.02002. Available online at: https://arxiv.org/abs/1708.02002. 10.1109/TPAMI.2018.2858826 30040631

[B14] Maisano F. Franzen O. Baldus S. Schäfer U. Hausleiter J. Butter C. (2013). Percutaneous mitral valve interventions in the real world: early and 1-year results from the ACCESS-EU, a prospective, multicenter, nonrandomized post-approval study of the MitraClip therapy in Europe. J. Am. Coll. Cardiol. 62 (12), 1052–1061. 10.1016/j.jacc.2013.02.094 23747789

[B15] Munafò R. Saitta S. Ingallina G. Denti P. Maisano F. Agricola E. (2024). A Deep Learning-Based Fully Automated Pipeline for Regurgitant Mitral Valve Anatomy Analysis From 3D Echocardiography. IEEE Access, 12, 5295–5308. 10.1109/ACCESS.2024.3349698

[B16] Munafò R. Saitta S. Tondi D. Ingallina G. Denti P. Maisano F. (2025). Automatic 4D mitral valve segmentation from transesophageal echocardiography: a semi-supervised learning approach. Med. Biol. Eng. Comput. 10.1007/s11517-024-03275-w 39797996 PMC13633368

[B17] Nkomo V. T. Gardin J. M. Skelton T. N. Gottdiener J. S. Scott C. G. Enriquez-Sarano M. (2006). Burden of valvular heart diseases: a population-based study. Lancet 368 (9540), 1005–1011. 10.1016/S0140-6736(06)69208-8 16980116

[B18] Odena A. Dumoulin V. Olah C. (2016). “Deconvolution and checkerboard artifacts,” in Distill. 10.23915/distill.00003

[B19] Ouyang D. He B. Ghorbani A. Yuan N. Ebinger J. Langlotz C. P. (2020). Video-based AI for beat-to-beat assessment of cardiac function. Nature 580, 252–256. 10.1038/s41586-020-2145-8 32269341 PMC8979576

[B20] Ronneberger O. Fischer P. Brox T. (2015). U-Net: Convolutional networks for biomedical image segmentation. arXiv 1505.04597, 234–241. 10.1007/978-3-319-24574-4_28

[B21] Schlemper J. Oktay O. Shaap M. Heinrich M. Kainz B. Glocker B. (2019). Attention gated networks: learning to leverage salient regions in medical images. arXiv:1808.08114 53, 197–207. 10.1016/j.media.2019.01.012 PMC7610718 30802813

[B22] Stone G. W. Lindenfeld J. Abraham W. T. Kar S. Lim D. S. Mishell J. M. (2018). Transcatheter mitral-valve repair in patients with heart failure. N. Engl. J. Med. 379 (24), 2307–2318. 10.1056/NEJMoa1806640 30280640

[B23] Synapse (2025). Synapse.org. Available online at: https://www.synapse.org/Synapse:syn51186045/wiki/621356.

[B24] Taskén A. Berg E. Grenne B. Holte E. Dalen H. Stølen S. (2023). Automated estimation of mitral annular plane systolic excursion by artificial intelligence from 3D ultrasound recordings. Artif. Intell. Med. 144, 102646. 10.1016/j.artmed.2023.102646 37783546

[B25] Thomas N. Unsworth B. Ferenczi E. Davies J. E. Mayet J. Francis D. P. (2008). Intraobserver variability in grading severity of repeated identical cases of mitral regurgitation. Am. Heart J. 156, 1089–1094. 10.1016/j.ahj.2008.07.017 19033003

[B26] Votta E. Caiani E. Veronesi F. Soncini M. Montevecchi F. M. Redaelli A. (2008). Mitral valve finite-element modelling from ultrasound data: a pilot study for a new approach to understand mitral function and clinical scenarios. Philo Trans. R. Soc. A Math. Phys. Eng. Sci. 366, 3411–3434. 10.1098/rsta.2008.0095 18603525

[B27] Wifstad S. Kildahl H. Grenne B. Holte E. Hauge S. W. Sæbø S. (2024). Mitral valve segmentation and tracking from transthoracic echocardiography using deep learning. Ultrasound Med. Biol. 50, 661–670. 10.1016/j.ultrasmedbio.2023.12.023 38341361

[B28] Zamorano J. Badano L. Bruce C. Chan K. L. Gonçalves A. Hahn R. T. (2011). EAE/ASE recommendations for the use of echocardiography in new transcatheter interventions for valvular heart disease. Eur. Heart J. 32, 2189–2214. 10.1093/eurheartj/ehr259 21885465

