# Supplementary material for: Towards standardizing mitral transcatheter edge-to-edge repair with deep-learning algorithm: a comprehensive multi-model strategy
Source: Front Netw Physiol. 2025 Nov 25;5:1701758. doi: 10.3389/fnetp.2025.1701758 (PMC12685852; doi:10.3389/fnetp.2025.1701758)
Supplement: Supplementary file 1 [file Supplementaryfile1.docx]

**Supplementary material**

**Supplementary Figures and Legends:**

**Supplementary Figure 1**. Attention gate mechanism schema.

**
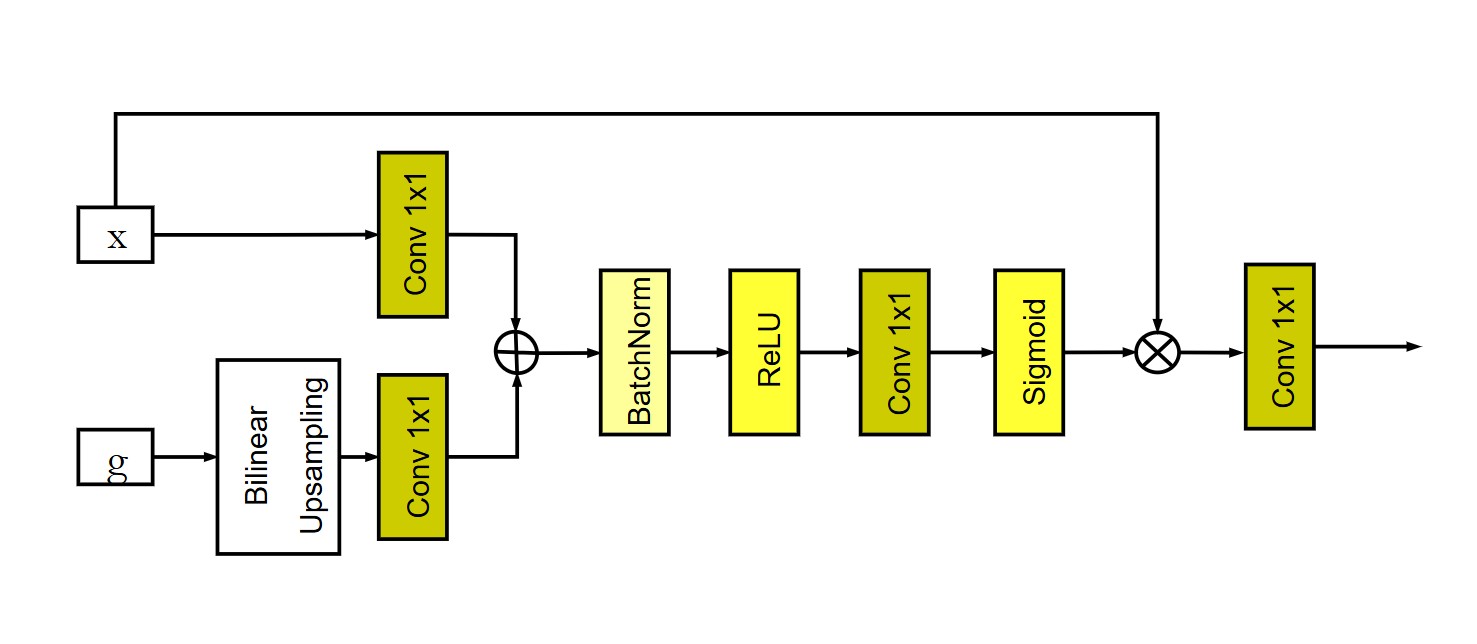
**

**Supplementary Figure 2**. Evaluation metrics used to assess the segmentation performance.

**Supplementary tables:**

| Supplementary Table 1 **Dataset distribution of TTE views** | | | |
| --- | --- | --- | --- |
| **Label** | **Train Set** | **Validation Set** | **Test Set** |
| apical_five_chamber | 825 frames 9 videos | - | 161 frames 3 videos |
| apical_four_chamber | 6,118 frames 51 videos | 3,210 frames  25 videos | 3,201 frames 26 videos |
| apical_long_axis | 3,312 frames  30 videos | 1,197 frames 8 videos | 1,930 frames 13 videos |
| apical_two_chamber | 3,700 frames 29 videos | 2,993 frames 23 videos | 2,622 frames 18 videos |
| plax | 5,392 frames 50 videos | 2,534 frames 19 videos | 2,160 frames 23 videos |
| psax_av | 1,401 frames 15 videos | 1,212 frames 14 videos | 991 frames 10 videos |
| psax_mv | 2,053 frames 17 videos | 2,073 frames 12 videos | 893 frames 10 videos |
| psax_pm | 1,143 frames  11 videos | 864 frames 6 videos | 592 frames 5 videos |
| subcostal | 699 frames 9 videos | 271 frames 3 videos | 437 frames 4 videos |
| subcostal_four_chamber | 1,275 frames 13 videos | 160 frames 2 videos | 670 frames 5 videos |
| suprasternal | 592 frames 6 videos | 147 frames 2 videos | 246 frames 2 videos |
| x_view | 138 frames 2 videos | - | 34 frames 1 video |
| other | 1,405 frames 20 videos | 918 frames 21 videos | 1,180 frames 13 videos |
| Total | 28,078 frames | 15,600 frames | 15,142 frames |
| AUC= area under curve; av= aortic valve; mv= mitral valve; pm= papillary muscle; TTE= transthoracic echocardiography; plax=parasternal long axis; psax=parasternal short axis. | | | |

| Supplementary Table 2 **Dataset distribution of TEE views** | | | |
| --- | --- | --- | --- |
| **Label** | **Train Set** | **Validation Set** | **Test Set** |
| 3_d | 3,724 frames 142 videos | 1,904 frames 70 videos | 1,704 frames 71 videos |
| av_short_axis | 3,506 frames 66 videos | 1,896 frames  31 videos | 2,017 frames 33 videos |
| bi_caval_view | 1,417 frames  21 videos | 502 frames 8 videos | 906 frames 9 videos |
| me_2_chamber | 1,710 frames 31 videos | 679 frames 14 videos | 1,136 frames 15 videos |
| me_4_chamber | 7,668 frames 119 videos | 3,374 frames 53 videos | 3,925 frames 57 videos |
| me_bicommissural | 5,240 frames 86 videos | 3,440 frames 54 videos | 2,858 frames 47 videos |
| mpr | 1,640 frames 56 videos | 900 frames 38 videos | 800 frames 32 videos |
| rv_inflow | 3,778 frames  58 videos | 1,027 frames 24 videos | 1,148 frames 27 videos |
| tg_long_axis | 197 frames 4 videos | 212 frames 4 videos | 140 frames 3 videos |
| tg_short_axis | 3,515 frames 47 videos | 2,085 frames 21 videos | 1,956 frames 23 videos |
| x_plan | 7,016 frames 145 videos | 3,730 frames 81 videos | 4,124 frames 75 videos |
| other | 12,711 frames 188 videos | 6,576 frames 98 videos | 6,211 frames 95 videos |
| Total | 62,539 frames 1,113 videos | 31,670 frames 581 videos | 31,317 frames 570 videos |
| AUC= area under curve; av= aortic valve; me= mid-esophageal; mpr= multiplanar reconstruction;rv=right ventricle; tg=transgastric; lvot=left ventricular outflow tract; 3_d= three-dimensional; TEE=transesophageal echocardiography. | | | |
